# Alcohol, stem cells and cancer

**DOI:** 10.18632/genesandcancer.156

**Published:** 2017-09

**Authors:** Shoujun Gu, Bao-Ngoc Nguyen, Shuyun Rao, Shulin Li, Kirti Shetty, Asif Rashid, Vivek Shukla, Chu-Xia Deng, Lopa Mishra, Bibhuti Mishra

**Affiliations:** ^1^ Department of Surgery, Center for Translational Medicine, George Washington University, Washington, DC, USA; ^2^ Departments of Pediatrics, The University of Texas MD Anderson Cancer Center, Houston, Texas, USA; ^3^ Division of Gastroenterology and Hepatology, Johns Hopkins University School of Medicine, Baltimore, Maryland, USA; ^4^ Departments of Gastroenterology and Liver Pathology, The University of Texas MD Anderson Cancer Center, Houston, Texas, USA; ^5^ Thoracic and Gastrointestinal Oncology Branch, National Cancer Institute, Bethesda, Maryland, USA; ^6^ Faculty of Health Sciences, University of Macau, Macau SAR, China; ^7^ Surgical Service, Veterans Affairs Medicale Center, Washington DC, USA

**Keywords:** alcohol, cancer, stem cells, TGF-β, DNA repair

## Abstract

Dosage, gender, and genetic susceptibility to the effects of alcohol remained only partially elucidated. In this review, we summarize the current knowledge of the mechanisms underlying the role of alcohol in liver and gastrointestinal cancers. In addition, two recent pathways- DNA repair and TGF-β signaling which provide new insights into alcohol in the regulation of cancers and stem cells are also discussed here.

## INTRODUCTION

Normal processing of endogenous as well as environmental factors and agents such as alcohol, is central to cellular homeostasis, and the deregulation of these pathways leads to liver and gastrointestinal inflammation and injury- often caused by a complex interplay between genetic and environmental factors [1]. In addition, protection from agents such as aldehydes generated spontaneously during cell metabolism is vital to normal cell function and tumor suppression [2, 3]. Until recently, dosage, gender and genetic susceptibility to the effects of alcohol remained only partially elucidated. For instance, women are known to be more susceptible than men, yet the specific populations and the underlying genetic mechanisms of alcohol susceptibility remain unclear [4, 5]. Multiple studies have examined the role of alcohol as a causative agent in multiple cancer types, that include breast, colon, esophageal, prostate and others [1, 6-9]. Yet few have been conducted prospectively, and the role of alcohol and its dosage in liver and gastrointestinal cancer is not completely understood [10, 11].

Although alcohol and illicit drugs are considered as different contributors to the global burden of disease, combined use of these substances is not an uncommon practice. Studies showed positive correlations between the incidence of alcohol and drug use [12-15]. A recent IARC (International Agency for Research on Cancer) meta-analysis examining the risk of esophageal cancer revealed the highest risk to be among those with concurrent alcohol and tobacco users, whereas the risk of esophageal cancer in alcohol users in the absence of tobacco usage was relatively lower [16]. Nevertheless, the risk of cancer is proportional to the dosage of used alcohol as many studies showed that compared to nondrinkers and occasional drinkers, the pooled relative risk (RR) was 1.03 for any, 0.97 for light, 1.04 for moderate, and 1.21 for heavy drinkers. As a matter of fact, the RR for heavy drinkers compared to nondrinkers and occasional drinkers was 5.13 for oral and pharyngeal cancer, 4.95 for oesophageal squamous cell carcinoma, 1.44 for colorectal, 2.65 for laryngeal and 1.61 for breast cancer. Heavy drinkers also had a significantly higher risk of cancer of the stomach, gallbladder, pancreas and lung [17]. In liver cancer, compared to nondrinker, the pooled RR was 0.91 for moderate drinking and 1.16 for heavy drinking [18]. Furthermore, for heavy drinkers, the pooled estimate RR was apparently higher for men than for women [19].

Alcohol is metabolized by alcohol dehydrogenases (ADHs) and aldehyde dehydrogenases (ALDHs). A review of case controlled studies examining genetic polymorphisms of the genes encoding these enzymes reveal a significant association between esophageal cancer risk and low *ADH1B* and *ALDH2* genotypes, especially in east Asian heavy drinkers [20, 21]. However, specific populations of high-risk individuals have yet to be clearly defined. Two recent pathways- one in DNA repair pathway and the other in TGF-β signaling have provided new insights that we discuss more broadly here [22, 23].

## ALCOHOL AND DNA REPAIR PATHWAYS

Since the discovery of DNA structure over 50 years ago, over 700 proteins and 900 distinct phosphorylation events have been described in the DNA damage response, reflecting the massive investment, cells make to preserve genomic integrity [24, 25]. When one considers, that ultraviolet light from sunlight induces up to 105 DNA lesions per cell per day, then the need for sensors is enormous. The repair mechanism involves at least six pathways that cover the specific steps involved in multiple DNA lesions [26, 27]. One of the most formidable of post replication DNA lesions, is the replication fork lesion, a barrier to chromosome duplication, which leads to mitotic catastrophe, complex chromosome rearrangements, and cell death. These lesions are managed by inter-strand cross link (ICL) repair systems to prevent replication fork progression [28]. The central components of the incisional and trans-lesional synthesis steps of the ICL system are the Fanconi complex, an E3 ligase, and at least four other factors. Fanconi anemia is a cancer pre-disposition syndrome characterized by hypersensitivity to DNA inter-strand cross-linking agents [29].

The thirteen Fanconi anemia(FA) complementation members act in a common pathway that result in DNA repair by homologous recombination. Replication-dependent ICL repair involves nucleolytic incisions flanking the ICL on one strand, trans-lesional DNA synthesis across the unhooked ICL, removal of the ICL by additional incisions, and homologous recombination. The central complex in this pathway is formed by the Fanconi anemia complementation group D2 (Fancd2), a core component of the Fanconi anemia complex, and FAI (FANCI) proteins forming the FANC1-FANCD2 (ID) complex that are phosphorylated by ATR (ataxia telangiectasia and Rad3-related). FA is caused by bi-allelic mutations of fifteen members of FANC pathway with inability to respond to cellular stress and ensuing DNA damage during S phase and loss of genome integrity [30, 31]. Patients with FA frequently develop bone marrow failure requiring allogeneic hematopoietic stem cell (HSC) transplantation, and have developmental abnormalities (short stature, a triangular face, and thumb abnormalities) [31, 32]. They also have a high risk of developing myelodysplasia (MDS), acute myeloid leukemia (AML) [31, 33-36], and hepatocellular carcinoma (HCC), especially with androgen treatment [37]. However, FANCD2 knockout mice do not develop HCC. Therefore, while the Fanconi anemia pathway is implicated in maintaining hematopoietic stem cell homeostasis, its role in liver stem cells and cancer remains unclear.

The development of cancer due to the failure of response to agents such as alcohol producing reactive aldehydes, creating adducts that directly bind and damage DNA, has recently been observed in models with genetic inactivation of the Fanconi anemia members [23]. Fanc mutant intercrosses with *ALDH2* mutant mice are susceptible to ethanol teratogenicity and defective DNA inter-strand cross link repair [23, 38]. Yet, mice with Fanc mutants (on their own) treated with alcohol do not develop any fetal-alcohol like aberrations suggesting a more complex process is involved in toxin induced DNA damage [2, 39-41] and that the essential sensors and the mechanisms for aberrant DNA damage from alcohol remain unclear.

## ALCOHOL AND TGF-Β PATHWAY

**Figure 1:**
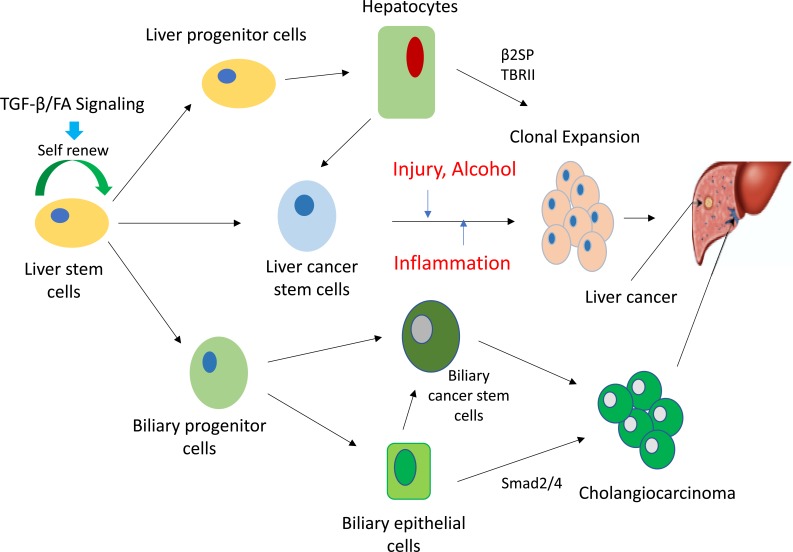
Schematic diagram of TGF-β/FA pathway dependent regulation on liver stem cells

TGF-β and the Fanconi anemia pathway, two critical pathways involved in both stem cell maintenance as well as differentiation, have also been shown to play a pivotal role in metabolizing alcohol. TGF-β serves as an essential regulator of cell polarity, growth, differentiation, and lineage specificity as well as a tumor suppressor pathway in multiple cell types [42, 43]. Defective TGF-β signaling is implicated in liver injury, inflammation and multiple cancers owing to the frequent somatic mutations in, or deregulation of, its components, such as Smad3, Smad4, and TGF-β receptors 1 and 2 (TBR1 and TBR2) (Figure 1). Smads are the intracellular mediators of TGF-β signaling [44-47], and their function is modulated by adaptor proteins such as the Smad anchor for receptor activation, filamin, microtubules, and β2-spectrin (β2SP, gene *Sptbn1*) [48-50].

TGF-β-activated Smads also orchestrate specific histone modifications and chromatin remodeling to activate their transcriptional targets. Keratinocytes cultured from TGFβ1-null mice have marked genomic instability that could accelerate tumor progression [51]. More recently, studies in the Smad4 conditional knockout mice that develop head and neck cancers, demonstrate a key role for Smad4 as a guardian of the genome through regulation of the Fanconi anemia/Brca (Fanc/Brca) DNA repair pathway [52, 53].

**Figure 2:**
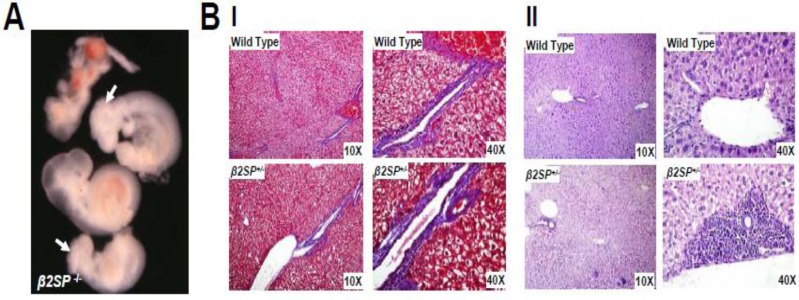
**A.** β2SP^−/−^ mouse embryos display some symptom of human fetal alcohol syndrome, microcephaly (white arrow), anencephaly and anophthalmia; **B**. Alcoholic hepatitis is induced by alcohol in β2SP^+/−^ mice compared to wild type normal controls. Alcoholic hepatitis-like picture in β2SP mutant mice: effect of alcohol on β2SP^+/−^ mice liver. Mice were treated with alcohol at dose of 30ml/day. I) Masson's Trichrome stain for alcohol treatment group; II) H&E stain for alcohol treatment group.

The major role of β2SP in maintaining genomic stability following alcohol-induced DNA damage is supported by the fact that β2SP defective mouse embryos display some symptom of human fetal alcohol syndrome (Figure 2) [22]. Furthermore, the development of hepatocellular cancers (HCCs) in β2SP heterozygote mutants establishes β2SP as a functional tumor suppressor. As a matter of fact, β2SP have been observed to associate with Fanconi proteins (G and D) as well as with DNA inter-strand cross-links [23, 41]. In addition, Smads have been identified as regulators of Fanconi genes [54]. Similarly, by virtue of its involvement in Smad3/4 localization and subsequent activation of Smad3/4, β2SP may enhance TGF-β tumor suppressor function. Indeed, loss of β2SP leads to decreased Fancd2 levels and sensitizes β2SP mutants to DNA damage by ethanol treatment, leading to phenotypes that closely resemble those observed in animals lacking both *ALDH2* and Fancd2 and resemble human fetal alcohol syndrome. *Sptbn1*-deficient cells are hypersensitive to DNA crosslinking agents and have defective DNA double-strand break repair that is rescued by ectopic Fancd2 expression. Taken together, TGF-β/β2SP signaling acts as a potential guardian of genomic stability from genotoxic metabolites through modulation of the Fanconi anemia DNA repair pathway, yet the exact mechanisms remain to be elucidated.

## ALCOHOL AND TLR4

TLR4 is a receptor for endotoxin which participates in many inflammatory processes such as M1 activation of hepatic macrophages in alcoholic liver disease [55]. However, its role in liver carcinogenesis via ectopic expression and activation has only recently been revealed in alcohol/HCV-associated HCC models [55]. Alcohol feeding to mice expressing the HCV Ns5a in a hepatocyte specific manner aggravates liver inflammation via activation of overexpressed TLR4 in the parenchymal cells [56]. Long-term alcohol feeding produces liver tumors in these transgenic mice in a manner dependent on TLR4. From these mice, tumor-initiating stem cell-like cells (TICs) have been isolated. These TICs exhibit self-renewal and tumorigenic activities driven by TLR4-dependent upregulation of the stem cell factor NANOG. A defective TGF-β tumor suppressor pathway is identified in the TICs and mediated by NANOG target genes IGF2BP3 and YAP1. Conversely, mice with an attenuated TGF-β pathway due to haploinsufficiency of β2- Spectrin, spontaneously develop liver tumors and alcohol feeding increases tumor incidence in a TLR4-dependent manner [57]. This reciprocal antagonism between TLR4 and TGF-β pathways may serve as a novel therapeutic target for HCC.

Whilst the phenotypes in the TGF-β deficient mutant mice are dramatic and restricted to specific compartments [58-60], it is clear that multiple tiers of control are present in the human disease- LOI at chromosome 11 leading to raised levels of TERT, IGF2, etc. The heterozygous TGF-β deficient mutants develop cancers spontaneously on a C57BL/6 background and in this regard the heterozygotes resemble sporadic cancer formation in humans [61-63]. There are limitations to all models, but over the last few years many of these have been selected and have been continually refined to optimize readouts. This has been successful to date and enabled the identification of new fundamental elements of two-tier control [64-66]. Finally, there are well established models of tumorigenesis available to study cancer progression in mice, as well as xenografts of cancer cell lines into recipients that many scientists have been able to employ successfully [57, 67].

## CONCLUSION

Collectively, the alcohol dosage and specific risk factors of alcohol use as the underlying cause of cancer remain unclear. Therefore, there is an urgent need for strong fundamental and clinical studies to examine the specific dosage, sex and genetic risk factors that confer cancer risk to alcohol intake.

## Abbreviations

IARC: International Agency for Research on Cancer
RR: Relative Risk
ADH: Alcohol Dehydrogenases
ALDH: Aldehyde Dehydrogenase
ICL: Interstrand Cross Link
HSC: Hematopoietic Stem Cell
MDS: Myelodysplasia
AML: Acute Myeloid Leukemia
HCC: Hepatocellular Carcinoma
TGF-β: Transforming Growth Factor beta
β2SP: β2-spectrin
Fanc: Fanconi Anemia
LOI: Loss of Imprint
TLR4: Toll-like Receptor 4
